# Mechanistic Insights into Phytocompounds for Vitiligo Therapy: Current Evidence and Future Opportunities

**DOI:** 10.3390/antiox15070863

**Published:** 2026-07-10

**Authors:** Rethabile Banda-Lesole, Ipeleng Kopano Rosinah Kgosiemang, Tshepiso Jan Makhafola

**Affiliations:** 1 Centre for Quality of Health and Living (CQHL), Faculty of Health and Environmental Sciences, Central University of Technology, Free State, Bloemfontein 9301, South Africa; 07rbanda@gmail.com (R.B.-L.); ikgosiemang@cut.ac.za (I.K.R.K.); 2 Stellenbosch Institute for Advanced Study (STIAS), Wallenberg Research Centre, Stellenbosch University, Stellenbosch 7600, South Africa; 3 Hamburg Institute for Advanced Study (HIAS), Rothenbaumchaussee 45, 20148 Hamburg, Germany

**Keywords:** vitiligo, phytocompounds, melanogenesis, oxidative stress, NRF2/ARE pathway, medicinal plants, redox signaling, antioxidant therapy

## Abstract

Vitiligo is a multifactorial depigmentation disorder involving complex interactions among oxidative stress, immune dysregulation, inflammatory signaling, and programmed cell death pathways, which act as a central driver of melanocyte dysfunction and loss, interacting with immune-mediated cytotoxicity and intrinsic cellular susceptibility. Excessive reactive oxygen species (ROS) disrupt mitochondrial integrity, impair redox homeostasis, suppress microphthalmia-associated transcription factor (MITF)-dependent melanogenesis, and induce melanocyte apoptosis. Concomitant dysfunction of the nuclear factor erythroid 2-related factor 2 (NRF2)/antioxidant response element (ARE) axis further exacerbates oxidative injury by limiting endogenous antioxidant capacity. Current therapeutic approaches, including corticosteroids, phototherapy, and targeted immunomodulators, achieve partial repigmentation but do not adequately resolve melanocyte-intrinsic redox imbalance. This structured narrative review comprehensively integrates mechanistic and translational evidence to define phytocompounds as redox-active, multi-target modulators in vitiligo. Plant-derived polyphenols, flavonoids, terpenoids, and related metabolites are shown to attenuate ROS accumulation, preserve mitochondrial function, activate NRF2-dependent antioxidant signaling, and restore MITF-mediated expression of tyrosinase and associated melanogenic enzymes. Furthermore, coordinated modulation of MAPK, PI3K/Akt, and JAK/STAT pathways highlights their capacity to regulate immune–oxidative crosstalk and promote melanocyte survival. Despite promising preclinical and emerging clinical evidence of repigmentation efficacy, translational progress remains limited by poor phytochemical standardization, insufficient transcriptional and proteomic validation, suboptimal stability and dermal bioavailability, and a lack of rigorously designed clinical trials. Collectively, this review provides a mechanistic framework linking redox dysregulation to melanocyte failure and positions phytocompounds as rational candidates for adjunctive or stand-alone antioxidant-based therapies, while defining critical priorities for clinical translation.

## 1. Introduction

Vitiligo is a chronic autoimmune depigmenting disorder characterized by the selective destruction of melanocytes, resulting in well-defined depigmented macules and patches on the skin and mucosal surfaces. The condition affects approximately 2% of the global population and occurs across all ethnic groups [1]. Clinically, vitiligo presents in localized or generalized forms, with lesions commonly appearing on the face, hands, feet, and other exposed areas of the body. Depigmentation may also affect hair follicles, leading to leukotrichia, and can affect melanocyte-containing structures of the eyes, including the uveal tract, potentially contributing to photosensitivity and ocular abnormalities. Beyond its dermatological manifestations, vitiligo is associated with a considerable psychological and social burden, including reduced quality of life, anxiety, depression, low self-esteem, and social stigma [1,2].

Vitiligo is currently recognized as a multifactorial disorder arising from complex interactions among genetic susceptibility, oxidative stress, immune dysregulation, and intrinsic melanocyte defects. At the molecular level, oxidative stress functions as a contributory upstream driver of melanocyte dysfunction and impaired melanogenesis. Excessive production of reactive oxygen species (ROS) disrupts redox homeostasis, leading to mitochondrial dysfunction, DNA damage, lipid peroxidation, protein oxidation, and activation of apoptotic pathways in melanocytes [3]. Melanocytes are particularly vulnerable to oxidative injury because melanin synthesis itself generates ROS as metabolic by-products. Under physiological conditions, antioxidant defense systems neutralize these reactive species; however, in vitiligo, impaired antioxidant capacity results in persistent oxidative stress and progressive melanocyte damage. Dysregulation of melanogenic regulators, particularly microphthalmia-associated transcription factor (MITF), further compromises melanocyte survival and pigment production by reducing the expression of key melanogenic enzymes such as tyrosinase, tyrosinase-related protein-1 (TRP-1), and tyrosinase-related protein-2 (TRP-2), thereby contributing to depigmentation of the skin and ocular tissues [4].

In recent years, increasing evidence has demonstrated that vitiligo is not solely a melanocyte-centered disorder but also involves dysfunction of the epidermal microenvironment, particularly keratinocytes [4,5]. Keratinocytes and melanocytes exist in a highly coordinated functional unit within the epidermis, where keratinocytes provide structural support and regulate melanocyte proliferation, differentiation, adhesion, survival, and melanogenesis through direct cell–cell contact and the secretion of growth factors and cytokines [5]. This dynamic melanocyte–keratinocyte crosstalk is essential for maintaining epidermal pigmentation and homeostasis. Under physiological conditions, keratinocytes produce melanocyte-supportive factors including stem cell factor (SCF), endothelin-1 (ET-1), basic fibroblast growth factor (bFGF), α-melanocyte-stimulating hormone (α-MSH), and various cytokines that promote melanocyte survival and melanin production [6].

However, oxidative stress profoundly alters keratinocyte function. Exposure to elevated ROS levels induces mitochondrial dysfunction and activates stress-responsive signaling pathways, including nuclear factor-kappa B (NF-κB), p38 mitogen-activated protein kinase (p38 MAPK), and inflammasome-associated pathways. Consequently, stressed keratinocytes release increased levels of pro-inflammatory cytokines such as tumor necrosis factor-alpha (TNF-α), interleukin (IL)-1β, IL-6, and IL-8, as well as chemokines including CXCL9, CXCL10, and CXCL16. These mediators contribute to the recruitment and activation of autoreactive CD8^+^ T cells and amplify local inflammatory responses that promote melanocyte destruction. In addition, oxidative stress reduces the production of melanocyte-supportive growth factors and disrupts adhesion molecules such as E-cadherin and integrins, impairing melanocyte attachment to neighboring keratinocytes and the basement membrane. Loss of adhesion promotes melanocytorrhagy, a process characterized by melanocyte detachment and subsequent elimination from the epidermis. Thus, keratinocyte dysfunction not only contributes to inflammatory signaling but also directly compromises melanocyte viability and epidermal homeostasis [7].

The interplay between oxidative stress and immune activation is now considered a fundamental pathogenic mechanism in vitiligo. Oxidative stress-induced damage leads to the release of damage-associated molecular patterns (DAMPs), heat shock proteins, and other danger signals that activate innate immune responses. These events stimulate dendritic cells and promote the production of interferon-gamma (IFN-γ), which activates the Janus kinase/signal transducer and activator of transcription (JAK/STAT) pathway. Subsequent induction of chemokines, particularly CXCL9 and CXCL10, facilitates the recruitment of autoreactive CD8^+^ cytotoxic T cells into lesional skin, where they target and destroy melanocytes [8]. Emerging evidence suggests that oxidative stress may act upstream of immune activation, creating a pathogenic microenvironment that initiates and perpetuates melanocyte-directed autoimmunity. Consequently, oxidative stress, keratinocyte dysfunction, and immune-mediated cytotoxicity form an interconnected pathogenic network that drives disease progression.

Conventional treatments aim primarily to suppress immune activity or stimulate melanocyte regeneration. Topical corticosteroids, topical calcineurin inhibitors, and phototherapy modalities such as psoralen–ultraviolet A (PUVA) and narrowband ultraviolet B (NB-UVB) remain first-line therapeutic options for localized and generalized vitiligo [9,10]. Corticosteroids suppress inflammatory and autoimmune responses directed against melanocytes, whereas calcineurin inhibitors such as tacrolimus and pimecrolimus inhibit T-cell activation and inflammatory cytokine production, thereby promoting repigmentation [11]. Phototherapy stimulates melanocyte proliferation, migration, and melanogenesis, facilitating repopulation of depigmented skin from residual melanocyte reservoirs within hair follicles [10]. More recently, Janus kinase (JAK) inhibitors have emerged as targeted immunomodulatory therapies capable of interrupting IFN-γ-mediated JAK/STAT signaling pathways involved in melanocyte destruction, representing a significant advancement in vitiligo management [12,13,14]. Despite these advances, current therapies remain largely symptomatic and are frequently limited by variable efficacy, prolonged treatment duration, disease recurrence, potential adverse effects such as skin atrophy and photosensitivity, and concerns regarding long-term safety [15]. These limitations underscore the need for therapeutic approaches that target the underlying mechanisms of oxidative stress, immune dysregulation, keratinocyte dysfunction, and melanocyte survival.

Consequently, there is growing interest in medicinal plants and phytochemicals as alternative or complementary therapeutic strategies for the treatment of vitiligo. Across African and Asian traditional medical systems, medicinal plants have long been used for the treatment of dermatological disorders, including pigmentation abnormalities. Species such as *Ginkgo biloba*, *Curcuma longa*, and *Psoralea corylifolia* have traditionally been employed to restore pigmentation and manage depigmenting skin conditions [16,17,18]. Increasing experimental evidence indicates that phytochemicals derived from these and other medicinal plants possess antioxidant, anti-inflammatory, immunomodulatory, and melanogenesis-promoting properties. Many of these compounds target key molecular pathways implicated in vitiligo, including NRF2-mediated antioxidant responses, NF-κB signaling, JAK/STAT activation, p38 MAPK signaling, and melanogenic regulators such as MITF and tyrosinase. Importantly, emerging studies suggest that phytochemicals may exert protective effects not only on melanocytes but also on keratinocytes by reducing oxidative stress, suppressing inflammatory cytokine production, preserving melanocyte–keratinocyte interactions, and restoring epidermal homeostasis [14,15].

In Southern African ethnomedicine, plant species such as *Helichrysum odoratissimum* and *Tagetes erecta* are widely used for the treatment of inflammatory and dermatological conditions. However, their mechanistic relevance to vitiligo pathogenesis remains largely unexplored. Given the remarkable biodiversity of African medicinal flora, systematic investigation of these ethnobotanical resources may reveal novel phytocompounds capable of modulating oxidative stress, inflammatory signaling, melanocyte survival, keratinocyte function, and melanogenic pathways [17,19,20]. Scientific validation of these traditional remedies is therefore essential for the development of evidence-based phytotherapeutic strategies and the discovery of novel anti-vitiligo agents [1,21]. A notable gap identified in the current literature is the under-representation of African medicinal plants in mechanistic, preclinical, and clinical studies despite their extensive ethnobotanical use and rich phytochemical diversity [22]. This review, therefore, uniquely integrates mechanistic, ethnobotanical, and translational perspectives, with particular emphasis on underexplored African medicinal plants as potential sources of novel therapeutics for vitiligo.

## 2. Medicinal Plants and Vitiligo Treatment: An Overview

**Figure 1:**
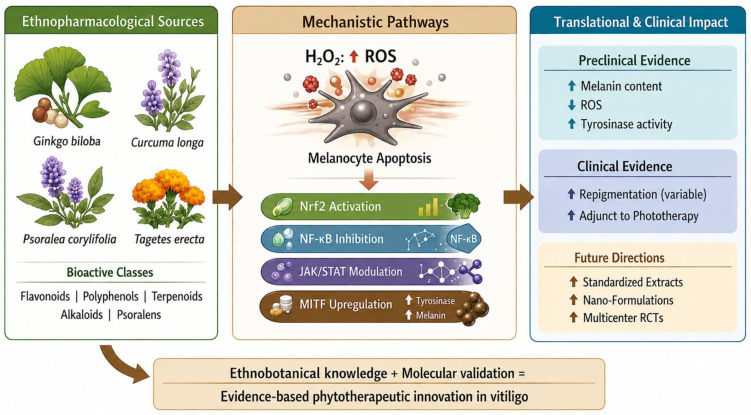
Conceptual framework of plant-derived phytocompounds in vitiligo therapy. Bioactive compounds from medicinal plants target key pathological mechanisms of vitiligo through antioxidant, anti-inflammatory, melanogenic, and cytoprotective activities, including modulation of NRF2, NF-κB, and JAK/STAT signaling pathways, ultimately promoting melanocyte survival and repigmentation.

Vitiligo remains a therapeutic challenge due to its multifactorial pathogenesis, which involves oxidative stress, immune dysregulation, and impaired melanogenesis [10,23]. Recent studies indicate that medicinal plant–derived phytocompounds may serve as promising adjuncts or alternatives to conventional therapies [24,25]. These phytochemicals encompass diverse classes, including flavonoids, polyphenols, terpenoids, alkaloids, and psoralens, each capable of modulating molecular pathways involved in melanocyte dysfunction (Figure 1) [26]. Through these multi-target mechanisms, phytocompounds may simultaneously mitigate oxidative damage, regulate inflammatory signaling, and stimulate melanogenesis, addressing the principal mechanisms driving vitiligo progression [27].

These identified compounds contribute to vitiligo management through multiple molecular mechanisms. At the mechanistic level, plant-derived compounds exert biological effects across several signaling pathways:

Oxidative Stress Reduction: By activating the nuclear factor erythroid 2-related factor 2 (NRF2) pathway and scavenging ROS, phytochemicals restore redox balance and protect melanocytes from oxidative damage [25].

Anti-Inflammatory Modulation: Regulation of inflammatory signaling pathways such as nuclear factor-kappa B (NF-κB) and Janus kinase/signal transducer and activator of transcription (JAK/STAT) reduces the production of cytokines involved in autoimmune-mediated melanocyte destruction [23,24].

Melanogenesis Stimulation: Upregulation of MITF and melanogenic enzymes, including tyrosinase, tyrosinase-related protein-1 (TRP-1), and tyrosinase-related protein-2 (TRP-2), enhances melanin biosynthesis and directly supports repigmentation [28].

Cytoprotection and Melanocyte Survival: Certain phytocompounds improve melanocyte resilience and survival under oxidative stress conditions, thereby preserving cellular viability and maintaining pigment-producing capacity [23]. Collectively, these molecular actions translate into clinically relevant outcomes, including antioxidant effects, reduced inflammation, and stimulation of repigmentation. The evidence underscores the multifaceted role of medicinal plants in vitiligo therapy, not only as sources of bioactive compounds but also as modulators of complex cellular networks. By simultaneously targeting oxidative, inflammatory, and melanogenic pathways, phytocompounds act as integrative agents capable of addressing the major mechanisms underlying vitiligo pathogenesis [10,25,26].

This review critically analyses the role of plant-derived phytocompounds in the management of vitiligo, focusing on their biological mechanisms, experimental evidence, therapeutic potential, and future research opportunities. The overarching goal of this review is to provide a critical synthesis of current evidence on the role of plant-derived phytocompounds in the management of vitiligo, integrating insights into their biological activity, mechanistic pathways, and therapeutic relevance [1,2]. Vitiligo is contextualized as a multifactorial depigmentation disorder characterized by oxidative stress, immune dysregulation, and melanocyte destruction, with significant clinical and psychosocial implications [2,10].

Major classes of phytocompounds, including polyphenols, flavonoids, alkaloids, and terpenoids, are comprehensively discussed alongside key medicinal plants implicated in pigmentation disorders. Particular emphasis is placed on the molecular mechanisms through which these compounds modulate redox homeostasis, inflammatory signaling, and melanogenesis pathways.

Available preclinical and clinical evidence has been critically evaluated to assess efficacy, safety, and translational potential. In parallel, persistent methodological limitations, such as variability in extract standardization, insufficient mechanistic validation, and limited clinical robustness, are highlighted as barriers to clinical application.

The review further outlines emerging research priorities, including the need for standardized phytochemical profiling, advanced in vitro and in vivo models, and well-designed clinical trials. Collectively, this synthesis positions phytocompounds as promising, yet under-optimized, candidates for the development of novel, mechanism-driven therapies for vitiligo.

## 3. Methodology

### 3.1. Review Design and Approach

This study was conducted as a structured narrative review to synthesize current evidence on medicinal plant-derived compounds for vitiligo therapy. Given the substantial heterogeneity in plant species, extraction methods, bioactive constituents, and experimental models (ranging from in vitro melanocyte cultures to animal studies and clinical investigations), a narrative synthesis approach was adopted to provide a comprehensive mechanistic overview rather than a quantitative meta-analysis [29]. While a narrative synthesis approach was adopted to provide a comprehensive mechanistic overview. Quality assessment of included studies was performed, using a literature search and study selection process, following a structured and transparent methodology. Specifically, the literature identification, screening, eligibility assessment, and study selection were conducted in accordance with the Preferred Reporting Items for Systematic Reviews and Meta-Analyses (PRISMA) 2020 guidelines to enhance transparency, reproducibility, and methodological rigor [30].

### 3.2. Literature Search Strategy

A systematic search was performed across primary electronic databases, including PubMed, Scopus, Web of Science, and ScienceDirect, from database inception to June 2026. To ensure comprehensive capture of relevant literature, Google Scholar was used as a supplementary resource, and manual screening of reference lists from eligible articles was performed.

Search terms combined keywords related to vitiligo, melanocyte biology, and phytochemicals. Example search strings included: vitiligo and medicinal plants, vitiligo and phytochemicals, melanocyte and plant extract, melanogenesis and phytocompounds, oxidative stress, melanocyte and natural products. Boolean operators (“AND”, “OR”) were used to refine the search strategy. Reference lists of relevant articles were also manually screened to identify additional studies.

### 3.3. Study Selection and Eligibility Criteria

All retrieved records were imported into the reference management software, namely Zotero 8.0.5 and Mendeley2.144.0. Duplicate entries were removed prior to screening, using the software’s duplicate detection function followed by manual verification to ensure accuracy. Titles and abstracts were screened to identify potentially relevant studies and articles that met the preliminary criteria, and were subsequently subjected to full-text evaluation.

Studies were included if they investigated medicinal plant extracts or plant-derived phytocompounds affecting melanocyte biology, melanogenesis, oxidative stress responses (e.g., NRF2/ROS), or inflammatory signaling (e.g., IFN-γ/JAK/STAT) in vitiligo or vitiligo-relevant experimental models. Eligible studies included peer-reviewed in vitro experiments, animal models, and clinical investigations published in English.

Exclusion criteria were applied to conference abstracts, editorials, and studies lacking primary experimental data or sufficient methodological detail.

The study selection process was documented using a PRISMA flow diagram to ensure transparency in the identification and inclusion of the relevant literature [29,30]. The PRISMA flow diagram summarizes the number of records identified, screened, excluded, assessed for eligibility, and ultimately included in the qualitative synthesis.

### 3.4. Data Extraction and Synthesis

Relevant data from eligible studies was systematically extracted, using a standardized data extraction framework. Extracted information included plant species used for identified phytochemicals or bioactive compounds, experimental model (e.g., melanocyte cell lines, oxidative stress model), key molecular pathways investigated (e.g., NRF2, MITF, tyrosinase), outcome measures (e.g., melanin content, ROS levels, tyrosinase activity, gene or protein expression), and key findings.

Due to heterogeneity in plant species, phytochemical composition, experimental models, and report outcomes, a narrative synthesis approach was employed [31] (Studies were grouped according to phytochemical class (e.g., flavonoids, polyphenols, terpenoids, alkaloids), and mechanistic pathways involved in melanocyte regulation, including oxidative stress modulation, inflammatory pathway regulation, and stimulation of melanogenesis.

## 4. Results

### 4.1. Study Selection PRISMA Flow

A structured and transparent literature search and study selection process was undertaken in accordance with the PRISMA 2020 guidelines to ensure transparency and reproducibility in study identification, screening, and selection [30]. As a result of the substantial heterogeneity across medicinal plant species, bioactive compounds, extraction methods, and experimental models, a narrative synthesis approach was adopted rather than a quantitative meta-analysis [31]. The methodological quality of the included studies was also critically appraised utilizing a predefined assessment criterion. A comprehensive search of selected electronic databases was complemented by manual screening of reference lists. The search strategy was developed using the Population–Intervention–Comparator–Outcome (PICO) framework, and the study selection process, including inclusion and exclusion decisions, is summarized in the accompanying PRISMA 2020 flow diagram, as can be seen below. The PRISMA 2020 flow diagram is used as a tool for transparent reporting and does not indicate this review as a full systematic review or meta-analysis [32].

**Figure 2:**
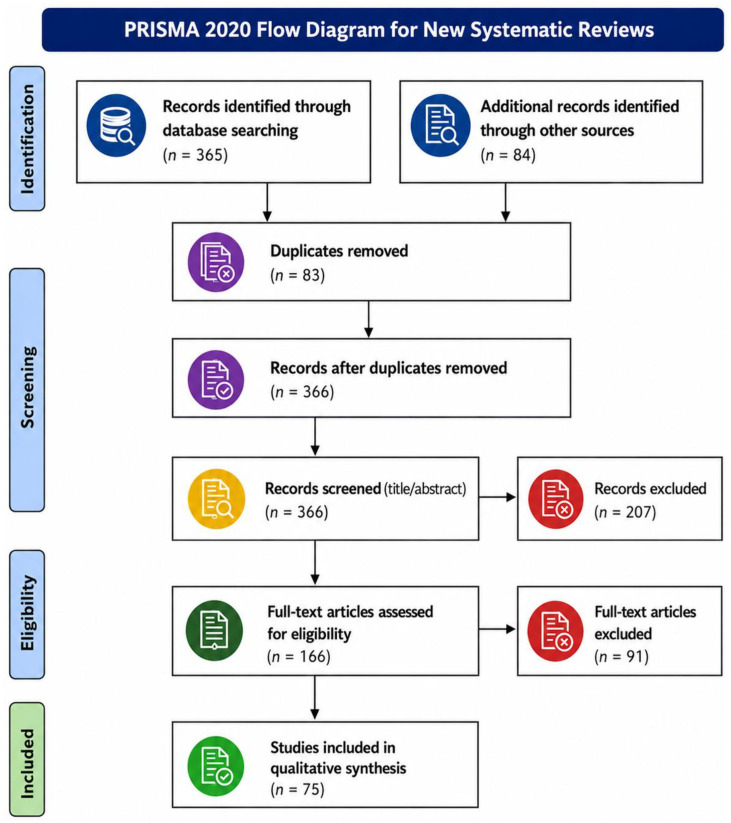
PRISMA 2020 flow diagram illustrating the structured search and study selection process for studies investigating medicinal plant-derived phytochemicals and therapeutic mechanisms relevant to vitiligo. The diagram summarizes the identification of records through database searching and additional sources, duplicate removal, title and abstract screening, full-text eligibility assessment, exclusion stages, and final inclusion of studies in the qualitative synthesis, in accordance with PRISMA 2020 reporting guidelines [30].

Database searching identified 365 records, with an additional 84 records identified through other sources (Figure 2). After removal of 83 duplicate records, 366 studies remained for title and abstract screening. Following screening, 207 records were excluded for being irrelevant to the review objectives. A total of 166 full-text articles were assessed for eligibility, of which 91 were excluded for not meeting the predefined inclusion criteria. Ultimately, 75 studies were included in the qualitative synthesis evaluating the mechanistic and therapeutic potential of medicinal plant-derived phytocompounds in vitiligo-related melanocyte dysfunction. An additional 8 references identified through citation tracking were included to support contextual and mechanistic interpretation, bringing the total number of included sources to 83.

The PRISMA flow below summarizes the identification, screening, eligibility, and inclusion steps.

### 4.2. Overview of Included Studies

The included studies primarily investigated the effects of plant-derived phytochemicals on melanocyte function under oxidative stress or inflammatory conditions. Most studies employed in vitro melanocyte or melanoma models, with a smaller number using animal or clinical models. Across the literature, there are several classes of plant-derived phytocompounds, including flavonoids, polyphenols, terpenoids, alkaloids, and psoralens, which exert multi-targeted biological effects relevant to vitiligo pathogenesis. Medicinal plants such as *Ginkgo biloba*, *Curcuma longa*, and *Psoralea corylifolia* were commonly evaluated, reflecting their traditional use in dermatological conditions [16,33,34].

Mechanistically, it has been revealed that these compounds influenced key pathogenic mechanisms associated with melanocyte dysfunction, including oxidative stress responses (ROS scavenging, NRF2 activation), inflammatory signaling (NF-κB, JAK/STAT inhibition), melanogenesis regulation (MITF, tyrosinase, TRP-1/2), and immunomodulation (T-cell and dendritic cell activity). These patterns highlight the multi-targeted biological potential of phytocompounds in vitiligo therapy, with certain species showing promising translational value for cytoprotection and repigmentation [35,36].

### 4.3. Classification of Phytocompounds Identified in the Reviewed Studies

Analysis of the included studies revealed flavonoids, polyphenols, terpenoids, alkaloids, and coumarins/psoralens as the major phytochemical classes involved in melanocyte protection and repigmentation. Flavonoids, including quercetin and rutin, were among the most frequently reported compounds and are abundant in plants such as *Ginkgo biloba*. These compounds exhibit antioxidant and anti-inflammatory properties, protecting melanocytes from oxidative stress-induced apoptosis and promoting cell survival [35,37]. Additionally, flavonoids have been reported to modulate immune-related signaling, contributing to the regulation of T cell and dendritic cell activity within the skin microenvironment [38]. Polyphenols, such as catechins from green tea and resveratrol from grapes, similarly provide antioxidative effects and influence immune responses implicated in vitiligo pathogenesis, including suppression of pro-inflammatory cytokines and modulation of NF-κB and JAK/STAT pathways [38,39]. Terpenoids, found in essential oils like lavender and tea tree, demonstrate anti-inflammatory and antimicrobial activities, supporting skin barrier integrity and reducing local inflammatory stress in pigmentary disorders [40]. Alkaloids, such as berberine, have been shown to enhance melanocyte proliferation, survival, and cytoprotection, while also modulating inflammatory pathways that contribute to melanocyte dysfunction [41]. Coumarins and psoralens, particularly derived from *Psoralea corylifolia*, function as photosensitizing agents that stimulate ultraviolet-induced melanogenesis. These compounds have been historically employed in photochemotherapy approaches for vitiligo treatment and can synergize with UV therapy to enhance repigmentation outcomes [2,22,36].

#### Strength of Evidence for Medicinal Plants in Vitiligo: Experimental Validation Versus Traditional Use

Among the medicinal plants investigated for vitiligo, the strongest evidence currently supports *Ginkgo biloba*, *Polypodium leucotomos*, *Cullen corylifolium* (bakuchi), *Ammi visnaga* (khellin source), *Picrorhiza kurroa*, *Piper nigrum*, *Curcuma longa*, and *Camellia sinensis*, which have demonstrated efficacy in in vitro, animal, and/or clinical studies [16]. In contrast, several traditionally used medicinal plants, including *Aloe vera*, *Azadirachta indica*, *Moringa oleifera*, *Withania somnifera*, *Sutherlandia frutescens*, *Hypoxis hemerocallidea*, and *Agave americana*, remain supported primarily by ethnomedicinal use and mechanistic plausibility, with limited vitiligo-specific experimental validation [32]. This disparity highlights a significant research gap and underscores the need for standardized phytochemical characterization, mechanistic investigations, and well-designed preclinical and clinical studies to substantiate traditional claims and identify novel anti-vitiligo therapeutics [42].

Key medicinal plants repeatedly investigated for bioactive compounds significant to vitiligo treatment include *Ginkgo biloba* (flavonoids, terpenoids); *Curcuma longa* (curcumin); *Psoralea corylifolia* (psoralens); *Helichrysum odoratissimum* (flavonoids, terpenoids, and phenolic compounds); *Tagetes erecta* (carotenoids, flavonoids, and terpenoids); *Camellia sinensis* (catechins); and *Vitis vinifera* (resveratrol) [42,43,44]. These plants have long-standing applications in dermatological disorders, and their phytochemicals exert complementary biological effects, including melanocyte protection, antioxidative and anti-inflammatory activities, immune modulation, and the promotion of melanogenesis, including photochemically induced pathways. Table 1 summarizes key medicinal plants, their bioactive compounds, and associated mechanisms identified across the reviewed studies.

### 4.4. Mechanistic Pathways of Phytocompounds in Melanocyte Protection

**Figure 3:**
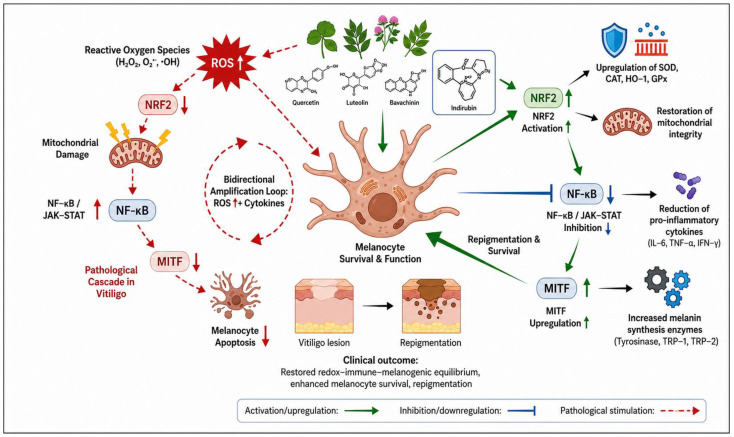
Mechanistic pathways of plant-derived phytocompounds in melanocyte protection within vitiligo pathology. The schematic representation of an integrated redox–immune–melanogenic framework illustrating how phytochemicals modulate key signaling pathways to restore melanocyte function.

Building on an integrated redox–immune–melanogenic and ethnopharmacological framework, plant-derived phytochemicals exert multi-targeted effects on melanocytes, influencing oxidative stress responses, inflammatory signaling, melanogenesis, and immune regulation, all of which are central to vitiligo pathogenesis [53]. Importantly, these mechanisms should not be interpreted as independent biological processes but rather as interconnected components of a unified redox–immune–melanogenic regulatory network [5,53]. Figure 3 summarizes the integrated mechanistic pathways through which phytocompounds protect melanocytes in vitiligo.

#### 4.4.1. Oxidative Stress and Redox Homeostasis

Oxidative stress is widely recognized as a central pathogenic factor in vitiligo, contributing to melanocyte dysfunction, apoptosis, and impaired melanogenesis. Elevated intracellular ROS in vitiligo-affected skin mediate melanocyte damage and disrupt cellular homeostasis [35]. Consequently, modulation of oxidative stress has emerged as a key therapeutic target in the development of plant-derived interventions for vitiligo [54].

A substantial body of experimental evidence indicates that phytocompounds with antioxidant properties can mitigate oxidative stress in melanocytes. Flavonoids, polyphenols, and terpenoids enhance cellular antioxidant defenses by activating the nuclear factor erythroid 2-related factor 2 (NRF2) signaling pathway, which upregulates cytoprotective enzymes such as superoxide dismutase, catalase, and heme oxygenase-1 [53,55]. NRF2 activation represents a central convergence point linking redox homeostasis, mitochondrial stability, and downstream preservation of melanogenic signaling.

Plant-derived extracts from *Ginkgo biloba*, *Curcuma longa*, *Helichrysum odoratissimum*, and *Tagetes erecta* have been specifically reported to reduce ROS accumulation and improve melanocyte survival in oxidative stress models [2,19,54].

#### 4.4.2. Inflammatory and Immune Dysregulation: The IFN-γ/CXCL10/CD8^+^ T-Cell Axis in Vitiligo

Vitiligo is increasingly recognized as a multifactorial autoimmune disorder where immune-mediated melanocyte destruction plays a central pathogenic role. Among the immunological pathways implicated in disease progression, the interferon-γ (IFN-γ)/CXCL10/CD8^+^ T-cell axis is one of the most extensively characterized mechanisms driving melanocyte loss [36,56]. Elevated levels of IFN-γ have been consistently detected in vitiligo lesions and patient sera, reflecting a pro-inflammatory microenvironment that promotes melanocyte dysfunction, apoptosis, and progressive depigmentation [23,25]. IFN-γ, produced primarily by activated CD8^+^ cytotoxic T cells, natural killer cells, and T helper 1 (Th1) lymphocytes, stimulates keratinocytes and other skin-resident cells to produce chemokines, particularly CXCL10. The binding of CXCL10 to its receptor CXCR3 facilitates the recruitment and retention of autoreactive CD8^+^ T cells within lesional skin. These T cells recognize melanocyte-associated antigens, including tyrosinase, gp100, and MART-1, and induce melanocyte death through perforin/granzyme release, Fas–Fas ligand interactions, and secretion of pro-inflammatory cytokines such as IFN-γ and tumor necrosis factor-α (TNF-α). The resulting IFN-γ/CXCL10 feedback loop perpetuates immune cell infiltration and sustains chronic melanocyte destruction [56,57]. Resident memory T (T_RM) cells further contribute to disease persistence and relapse by retaining immunological memory against melanocyte antigens and rapidly reinitiating inflammatory responses following clinical repigmentation [58].

Growing evidence indicates that oxidative stress functions upstream of immune activation in vitiligo. Excessive accumulation of reactive oxygen species (ROS) promotes melanocyte injury and the release of damage-associated molecular patterns (DAMPs), which enhance antigen presentation and stimulate adaptive immune responses. Consequently, oxidative stress and inflammation operate within a self-amplifying cycle in which ROS-induced cellular damage promotes immune activation, while inflammatory cytokines further exacerbate oxidative stress and melanocyte degeneration [59]. The NRF2 pathway serves as a critical molecular interface between these interconnected processes. By enhancing antioxidant defenses and reducing intracellular ROS accumulation, NRF2 activation restores redox homeostasis, mitigates oxidative damage, and indirectly suppresses NF-κB- and JAK/STAT-mediated inflammatory signaling. Through disruption of this oxidative–inflammatory feedback loop, NRF2 contributes to the preservation of melanocyte viability and maintenance of epidermal homeostasis [54].

Several phytocompounds exhibit both antioxidant and immunomodulatory properties capable of targeting these complementary pathogenic mechanisms. Experimental studies have demonstrated that flavonoids, polyphenols, and related bioactive compounds suppress the production of pro-inflammatory cytokines, including IFN-γ, IL-6, and TNF-α, while modulating NF-κB-, JAK/STAT-, and NRF2-dependent signaling pathways involved in melanocyte injury. By simultaneously restoring redox balance and attenuating immune activation, these phytochemicals may provide greater therapeutic benefit than single-target interventions [37]. Collectively, these findings underscore the importance of coordinated regulation of oxidative stress and immune responses in maintaining melanocyte survival and supporting repigmentation. However, current evidence remains predominantly preclinical, highlighting the need for mechanistic investigations and well-designed clinical studies evaluating both redox- and immune-related endpoints in vitiligo therapy [60].

#### 4.4.3. Cytoprotective Effects of Phytocompounds on Keratinocytes and Melanocytes

Although melanocyte loss is the defining pathological feature of vitiligo, increasing evidence indicates that disruption of melanocyte–keratinocyte crosstalk plays a critical role in disease initiation and progression. Under physiological conditions, keratinocytes maintain epidermal homeostasis by regulating melanocyte survival, proliferation, adhesion, and melanogenic activity through direct cell–cell interactions and the secretion of melanocyte-supportive factors, including stem cell factor (SCF), basic fibroblast growth factor (bFGF), endothelin-1 (ET-1), and α-melanocyte-stimulating hormone (α-MSH). These interactions are essential for maintaining melanocyte viability and normal pigment production [61].

In vitiligo, oxidative stress and inflammatory signaling disrupt these regulatory functions, leading to impaired epidermal homeostasis and progressive melanocyte degeneration. Damaged keratinocytes exhibit reduced production of trophic growth factors and diminished expression of adhesion molecules such as E-cadherin and integrins, compromising melanocyte attachment to neighboring keratinocytes and to the basement membrane. Loss of these adhesive interactions promotes melanocytorrhagy, a process characterized by melanocyte detachment and increased susceptibility to apoptosis and immune-mediated clearance [59]. Simultaneously, keratinocyte-derived inflammatory mediators, including TNF-α and IFN-γ, suppress melanogenic signaling pathways and further impair melanocyte proliferation and survival, creating a self-perpetuating cycle of epidermal dysfunction [36,62].

A consistent finding across experimental studies was that many phytocompounds exerted protective effects on both melanocytes and keratinocytes through antioxidant, anti-inflammatory, and cytoprotective mechanisms. Polyphenols, flavonoids, and related bioactive compounds preserve cellular redox homeostasis, maintain epidermal integrity, and protect against oxidative stress-induced mitochondrial dysfunction and apoptosis. By restoring a favorable epidermal microenvironment, these compounds help sustain melanocyte viability and support functional melanocyte–keratinocyte interactions [63].

Importantly, several phytochemicals have also been shown to stimulate melanogenic pathways. Enhanced expression of microphthalmia-associated transcription factor (MITF), tyrosinase, tyrosinase-related protein-1 (TRP-1), and tyrosinase-related protein-2 (TRP-2) has been reported following treatment with plant-derived bioactive compounds, resulting in increased melanin synthesis and improved melanocyte function [63,64]. Within the integrated pathogenic framework of vitiligo, activation of melanogenesis appears to occur largely as a downstream consequence of restored redox balance and attenuation of inflammatory signaling rather than as an isolated event. Through preservation of melanocyte viability, maintenance of keratinocyte support functions, and enhancement of MITF-dependent melanogenic pathways, phytochemicals may contribute to the restoration of pigmentation and epidermal homeostasis.

Collectively, these findings highlight that successful repigmentation depends not only on stimulating melanogenesis but also on preserving the functional interactions between melanocytes and keratinocytes. Therapeutic strategies capable of simultaneously restoring epidermal homeostasis, maintaining cellular adhesion, protecting against oxidative and inflammatory injury, and promoting melanogenic activity may therefore provide the greatest benefit for preventing melanocyte loss and supporting repigmentation in vitiligo.

#### 4.4.4. Ferroptosis

Recent advances in vitiligo research have identified ferroptosis as a potential contributor to melanocyte dysfunction and depletion [65]. Unlike apoptosis, ferroptosis is characterized by excessive lipid peroxidation and the accumulation of iron-dependent reactive lipid species that ultimately compromise membrane integrity and induce cell death. Thus, melanocytes are exposed to substantial oxidative and metabolic stress associated with melanogenesis, resulting in increased vulnerability to ferroptotic injury [66].

Current evidence suggests that ferroptosis represents a downstream consequence of the oxidative imbalance already recognized as a trademark of vitiligo. Persistent redox dysregulation can disrupt iron homeostasis, promote lipid peroxidation, and weaken antioxidant defense systems, thereby creating conditions favorable for ferroptotic cell death. Central to this process is the glutathione (GSH)–glutathione peroxidase 4 (GPX4) axis, which normally protects cells by detoxifying lipid hydroperoxides. Impairment of GSH availability or GPX4 activity reduces the capacity of melanocytes to neutralize lipid oxidative damage, increasing susceptibility to ferroptosis [67]. Experimental evidence further suggests that mitochondrial dysfunction and defective NRF2-mediated cytoprotective responses may exacerbate this vulnerability.

Ferroptosis may also contribute to disease progression beyond its direct effects on melanocyte survival. Cellular damage associated with ferroptosis death has the potential to amplify local inflammatory responses and reinforce pathogenic immune mechanisms already implicated in vitiligo, including the IFN-γ/CXCL10/CD8^+^ T-cell axis. Consequently, ferroptosis is increasingly viewed as a mechanistic bridge linking oxidative stress to immune-mediated melanocyte destruction rather than as an isolated pathway [58].

The growing recognition of ferroptosis has generated interest in phytochemicals capable of modulating ferroptosis-related pathways. Many polyphenols, flavonoids, phenolic acids, and terpenoids possess antioxidant, metal-chelating, and cytoprotective properties that may reduce susceptibility to ferroptosis injury. Through maintenance of intracellular redox balance and support of endogenous antioxidant systems, these compounds may help preserve GSH-dependent defenses, limit lipid peroxidation, and enhance melanocyte resilience. Phytochemicals such as curcumin, quercetin, apigenin, epigallocatechin gallate (EGCG), and resveratrol have demonstrated anti-ferroptosis effects in various experimental models through modulation of lipid oxidation, iron metabolism, and NRF2-associated cytoprotective pathways [68]. Although direct evidence in vitiligo remains limited, these findings suggest that inhibition of ferroptosis may represent an additional mechanism through which phytochemicals support melanocyte survival [69].

Collectively, ferroptosis is emerging as a novel component of the pathogenic network underlying vitiligo. Rather than functioning independently, it appears to intersect with established mechanisms involving oxidative stress, impaired antioxidant defenses, and immune-mediated melanocyte injury. Further investigation of ferroptosis-related biomarkers and therapeutic interventions may therefore provide new opportunities for preventing melanocyte loss and enhancing repigmentation outcomes [70].

#### 4.4.5. p38 MAPK as a Therapeutic Target

The p38 mitogen-activated protein kinase (p38 MAPK) pathway functions as a critical convergence point linking oxidative stress to inflammatory and apoptotic signaling in vitiligo. Activated by reactive oxygen species (ROS), inflammatory cytokines, and other cellular stressors, p38 MAPK promotes melanocyte dysfunction through interactions with transcription factors such as AP-1 and NF-κB, thereby amplifying inflammatory gene expression and perpetuating the oxidative–inflammatory microenvironment. Persistent activation of p38 MAPK also contributes to melanocyte apoptosis by inducing mitochondrial dysfunction, disrupting Bax/Bcl-2 balance, activating caspases, and suppressing melanogenic regulators, including microphthalmia-associated transcription factor (MITF), tyrosinase, tyrosinase-related protein-1 (TRP-1), and tyrosinase-related protein-2 (TRP-2). Consequently, p38 MAPK contributes to both melanocyte loss and impaired pigment production [71].

Given its central role in melanocyte injury, p38 MAPK has emerged as a promising therapeutic target. Numerous phytochemicals, particularly polyphenols and flavonoids, have demonstrated the ability to modulate p38 MAPK signaling while simultaneously enhancing antioxidant defenses and suppressing inflammatory responses [72]. Apigenin exemplifies this mechanism, having been shown to reduce intracellular ROS accumulation, enhance antioxidant enzyme activity, inhibit p38 MAPK and NF-κB activation, decrease pro-inflammatory cytokine production, and protect melanocytes from oxidative stress-induced apoptosis [61]. These findings support the broader concept that phytochemicals may preserve melanocyte viability through coordinated antioxidant, anti-inflammatory, and cytoprotective actions targeting key stress-responsive signaling pathways.

#### 4.4.6. Integrated Mechanistic Actions of Phytocompounds

Building on the individual mechanisms developed in Section 4.4.1, Section 4.4.2, Section 4.4.3, Section 4.4.4 and Section 4.4.5—mitochondrial dysfunction and ROS handling (Section 4.4.1), NRF2/ARE antioxidant activation (Section 4.4.2), NF-κB-driven inflammatory signaling (Section 4.4.3), IFN-γ/CXCL10/CXCR3^+^ CD8^+^ T-cell recruitment (Section 4.4.4), and p38 MAPK-mediated stress-apoptosis coupling (Section 4.4.5)—current evidence indicates that phytocompounds rarely modulate single pathways in isolation. Multi-target regulation instead appears to be a defining feature, with flavonoids, polyphenols, terpenoids, and psoralens converging on the three pathophysiological axes that sustain melanocyte destruction in vitiligo: redox imbalance, immune activation, and suppression of melanogenic signaling [73,74].

NRF2–NF-κB–JAK/STAT crosstalk provides a unifying mechanistic frame. NRF2 activation promotes transcription of cytoprotective enzymes, including heme oxygenase-1, catalase, and superoxide dismutase, attenuating oxidative damage and preserving melanocyte viability [55]. In parallel, suppression of NF-κB and JAK/STAT signaling, particularly the IFN-γ–driven JAK1/JAK2–STAT1 axis (developed in Section 4.4.3 and Section 4.4.4), reduces CXCL9/CXCL10-mediated recruitment of CXCR3^+^ CD8^+^ T cells and downstream melanocyte cytotoxicity [36,75]. Because oxidative stress amplifies inflammatory signaling through Damage-Associated Molecular Pattern release and enhanced antigen presentation (introduced in Section 4.4.1), restoring redox homeostasis indirectly attenuates adaptive immune activation, producing a self-reinforcing anti-inflammatory effect.

Convergence with melanogenic regulation (developed in Section 4.4.5) completes the integrated model. Compounds acting simultaneously on MITF/tyrosinase signaling and on redox/inflammatory pathways restore pigment-producing capacity alongside melanocyte survival. This integrative activity is consistent with systems-pharmacology models describing phytochemicals as regulators of interconnected signaling networks rather than isolated molecular targets [76].

#### 4.4.7. Clinical and Translational Evidence

Clinical investigations, particularly those involving extracts from *Ginkgo biloba* and psoralen-containing preparations derived from *Psoralea corylifolia*, have reported variable but generally promising repigmentation outcomes, especially when used as adjuncts to phototherapy [77]. However, interpretation of these findings is limited by small sample sizes, heterogeneity in study design, lack of standardized extract formulations, and relatively short follow-up periods. Despite these limitations, mechanistic consistency across in vitro, animal, and clinical studies supports a shared therapeutic framework based on oxidative stress reduction, immune modulation, and melanogenesis activation [78].

#### 4.4.8. Ethnopharmacological and Translational Perspective

Integration of these findings with ongoing mechanistic investigations involving *Helichrysum odoratissimum* and *Tagetes erecta* suggests that ethnobotanically prioritized medicinal plants rich in flavonoids, polyphenols, and terpenoids may exert cytoprotective, anti-inflammatory, and melanogenic effects in melanocytes exposed to oxidative and inflammatory stress conditions [19]. These observations highlight the importance of ethnopharmacological knowledge systems as a source of multi-target therapeutic candidates for the treatment of vitiligo.

### 4.5. Melanogenesis Promotion

A major therapeutic objective in vitiligo management is the restoration of melanocyte function and melanin synthesis. Experimental evidence indicates that several plant-derived phytocompounds promote melanogenesis through modulation of melanocyte differentiation and pigment-production pathways. Increased expression of melanogenic regulators, including microphthalmia-associated transcription factor (MITF), tyrosinase (TYR), tyrosinase-related protein-1 (TRP-1), and tyrosinase-related protein-2 (TRP-2), has been consistently associated with enhanced melanin biosynthesis and repigmentation potential [64].

In melanocyte and melanoma-based experimental systems, flavonoids and polyphenols have been reported to enhance melanin production, melanocyte dendricity, and melanosome transfer to keratinocytes, thereby supporting functional pigmentation recovery. Psoralen-containing compounds derived from *Psoralea corylifolia* further demonstrate clinically relevant melanogenic activity by enhancing ultraviolet-induced pigmentation responses during phototherapy [79,80]. Rather than functioning as isolated pigment-inducing agents, these phytocompounds appear to promote melanogenesis within the broader context of restored redox balance and reduced inflammatory stress, thereby supporting sustained melanocyte functionality and repigmentation.

Although melanogenesis-promoting effects have been widely reported, translational interpretation remains constrained by variability in phytochemical composition, dosing strategies, and experimental model systems. Further mechanistic validation using standardized melanocyte-based platforms will therefore be necessary to define reproducible therapeutic applications in vitiligo management [81,82].

### 4.6. Cytoprotective and Survival Effects

Experimental studies using vitiligo-relevant oxidative stress models demonstrate that multiple phytocompounds exert protective effects on melanocyte viability under pathological conditions. In hydrogen peroxide (H_2_O_2_)-induced melanocyte injury models, flavonoids, polyphenols, terpenoids, and alkaloids have been associated with preservation of mitochondrial integrity, improved cellular viability, and attenuation of oxidative stress–induced apoptosis [36,75].

These cytoprotective effects are frequently accompanied by reduced apoptotic signaling and maintenance of melanocyte metabolic activity, suggesting that phytocompounds may support melanocyte persistence during oxidative and inflammatory stress exposure. Preservation of viable melanocyte populations is particularly relevant in vitiligo, where progressive melanocyte loss contributes directly to depigmentation and impaired pigment recovery. Importantly, cytoprotection should be interpreted as a downstream functional consequence of integrated redox and inflammatory pathway modulation rather than an independent therapeutic mechanism. In this context, maintenance of melanocyte survival provides a biological foundation for subsequent melanogenic recovery and repigmentation.

Despite encouraging preclinical findings, most available evidence remains derived from in vitro experimental systems, highlighting the need for translational validation in standardized animal and clinical models.

### 4.7. Immunomodulatory Effects of Phytocompounds

Vitiligo is increasingly recognized as an immune-associated depigmenting disorder characterized by cytotoxic CD8^+^ T-cell activity, inflammatory cytokine production, and progressive melanocyte destruction [36,56,82]. Persistent inflammatory signaling within the cutaneous microenvironment contributes to melanocyte dysfunction and impaired pigmentation stability.

Several phytocompounds demonstrate immunomodulatory properties capable of attenuating inflammatory responses associated with vitiligo pathogenesis. Experimental evidence indicates that flavonoids, polyphenols, and related bioactive compounds suppress the production of pro-inflammatory cytokines, including interferon-γ (IFN-γ), interleukin-6 (IL-6), and tumor necrosis factor-α (TNF-α), while modulating immune-related signaling pathways implicated in melanocyte injury [37,38]. Within the integrated redox–immune framework described in Section 4.4, immunomodulatory activity is closely linked to oxidative stress regulation, reflecting the interconnected nature of inflammatory and redox-mediated melanocyte damage. Consequently, phytocompounds capable of simultaneously reducing inflammatory signaling and oxidative stress may provide greater therapeutic relevance than single-target interventions.

Current evidence supporting these immunomodulatory effects remains predominantly preclinical, emphasizing the importance of future mechanistic and clinical studies investigating immune-specific endpoints in vitiligo therapy.

#### Ferroptosis: A Novel Mechanistic Lin

Given the central role of redox imbalance in ferroptosis, antioxidant phytocompounds may offer therapeutic potential by targeting multiple steps within the ferroptotic cascade. Polyphenols, flavonoids, phenolic acids, and terpenoids possess potent free-radical scavenging and metal-chelating properties that can reduce ROS accumulation and limit iron-catalyzed lipid peroxidation. Several phytochemicals have also been shown to activate the NRF2 signaling pathway, enhancing the expression of antioxidant and cytoprotective genes involved in glutathione synthesis, iron homeostasis, and cellular detoxification. By restoring intracellular GSH levels, preserving GPX4 activity, and reducing lipid oxidative damage, these compounds may improve melanocyte resistance to ferroptotic injury [26,38].

Compounds such as curcumin, quercetin, apigenin, epigallocatechin gallate (EGCG), and resveratrol have demonstrated antioxidant, anti-inflammatory, and cytoprotective effects that could theoretically suppress ferroptosis-associated melanocyte death. Although direct evidence in vitiligo models remains limited, these phytochemicals have been shown in other disease contexts to inhibit lipid peroxidation, regulate iron metabolism, enhance glutathione-dependent antioxidant defenses, and modulate NRF2-mediated signaling pathways. Consequently, ferroptosis represents a promising therapeutic target through which phytocompounds may complement existing approaches aimed at reducing oxidative stress and preserving melanocyte survival [81].

### 4.8. Key Plants and Their Bioactive Compounds

**Figure 4:**
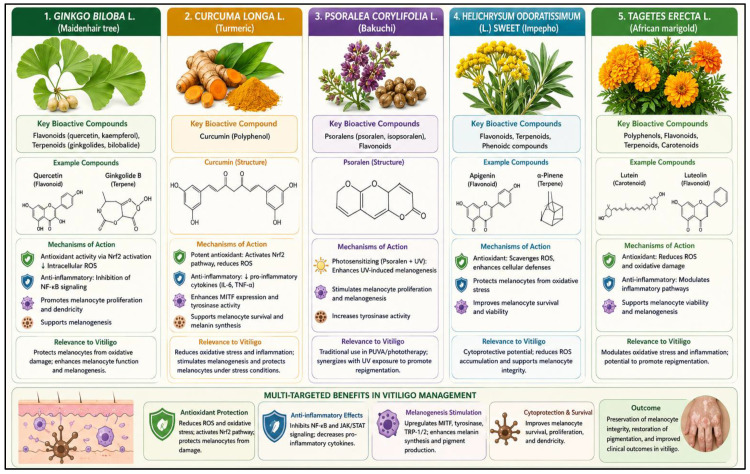
Ethnobotanically prioritized medicinal plants and their multi-targeted therapeutic potential in vitiligo management. The figure categorizes five key species—*G. biloba* L., *C. longa* L., *P. corylifolia* L., *H. odoratissimum* Sweet, and *T. erecta* L.—and their respective bioactive compounds (e.g., quercetin, curcumin, and psoralens). The diagram illustrates a multi-targeted mechanism of action where these phytocompounds address vitiligo pathophysiology by providing antioxidant protection (e.g., NRF2 activation), exerting anti-inflammatory effects (e.g., NF-κB inhibition), and stimulating melanogenesis through increased tyrosinase activity.

Several ethnobotanically prioritized medicinal plants have been repeatedly investigated for their bioactive compounds and therapeutic potential in the treatment of vitiligo. These plants are rich in flavonoids, polyphenols, terpenoids, and psoralens, which exert complementary mechanisms including antioxidant, anti-inflammatory, melanogenesis-stimulating, and cytoprotective effects. Figure 4 summarizes key medicinal plants, their major bioactive compounds, and their proposed mechanistic roles in vitiligo management.

*G. biloba* is a well-studied plant whose primary bioactive constituents are flavonoids and terpenoids. These compounds exhibit antioxidant and anti-inflammatory properties, reducing intracellular reactive oxygen species (ROS) and inhibiting inflammatory signaling pathways such as NF-κB [37,54]. Additionally, *G. biloba* extracts have been reported to promote melanocyte proliferation, enhance dendricity, and support melanogenic activity, making it a promising candidate for adjunctive vitiligo therapy. *C. longa* contains the polyphenolic compound curcumin, which demonstrates potent antioxidant, anti-inflammatory, and melanogenesis-stimulating effects [39]. Curcumin modulates oxidative stress through activation of the NRF2 pathway, attenuates inflammatory cytokine production, and has been shown to enhance MITF expression and tyrosinase activity in melanocyte models, supporting melanin synthesis and melanocyte survival under stress conditions. *P. corylifolia* is traditionally used in photochemotherapy for vitiligo due to its content of psoralen, a photosensitizing coumarin derivative. Psoralen compounds stimulate melanocyte proliferation and melanogenesis by synergizing with ultraviolet (UV) exposure, thereby enhancing repigmentation outcomes [23]. In combination with UV therapy, psoralen has demonstrated consistent clinical efficacy in promoting pigment restoration in vitiligo-affected skin. *H. odoratissimum* is another medicinal plant traditionally used in South African dermatology. Its flavonoid and terpenoid-rich extracts have been shown to protect melanocytes from oxidative stress, reduce ROS accumulation, and enhance melanocyte survival, highlighting its potential as a cytoprotective agent [19]. *T. erecta* contains polyphenols and flavonoids with antioxidant and anti-inflammatory properties. Experimental studies indicate that these compounds can modulate oxidative stress and inflammatory pathways in melanocytes, supporting cellular viability and potentially promoting repigmentation [19].

Collectively, these plants exemplify how bioactive phytocompounds can target multiple pathways implicated in vitiligo pathogenesis, including oxidative stress, inflammatory signaling, melanocyte apoptosis, and impaired melanogenesis. Their integration into mechanistically guided research strengthens the translational potential of traditional medicinal plants in modern dermatological therapeutics.

### 4.9. Evidence Specific to South African Medicinal Plants

The therapeutic potential of plant-derived phytocompounds for vitiligo has been evaluated across in vitro, animal, and clinical studies, providing complementary evidence of their mechanisms and efficacy.

#### 4.9.1. In Vitro Studies

Despite the extensive global literature on plant-derived therapies for vitiligo, evidence specifically addressing South African medicinal plants remains relatively limited. Ethnobotanical surveys from the region document numerous plant species traditionally used for dermatological conditions, yet only a small subset has undergone mechanistic investigation relevant to melanocyte biology or vitiligo pathogenesis. Several studies have investigated the effects of phytocompounds on melanocyte cultures, including primary human melanocytes and immortalized cell lines. These studies consistently demonstrate that flavonoids, polyphenols, terpenoids, and alkaloids enhance melanocyte proliferation, migration, and dendricity while promoting melanogenesis [37].

Phytochemicals also modulate oxidative stress and inflammatory pathways in vitro. For instance, flavonoid- and polyphenol-rich extracts reduce intracellular reactive oxygen species (ROS) accumulation and activate the NRF2-mediated antioxidant response, thereby protecting melanocytes from oxidative damage [19,55]. Concurrently, inhibition of NF-κB and JAK/STAT signaling pathways by these compounds reduces pro-inflammatory cytokine production, including IL-6 and TNF-α, mitigating inflammatory stress in melanocytes [36,56].

#### 4.9.2. Animal Models

Animal studies provide mechanistic and translational support for the repigmentation potential of phytocompounds. UV-induced depigmentation models in mice or guinea pigs have been employed to mimic vitiligo-like skin changes. Administration of extracts from *Ginkgo biloba*, *Curcuma longa*, and psoralen-containing *Psoralea corylifolia* in these models reduced depigmentation, enhanced melanocyte survival, and promoted repigmentation [2,22,56].

Mechanistically, these interventions were associated with reduced oxidative stress, increased expression of melanogenic markers (MITF, TYR, TRP-1, TRP-2), and modulation of inflammatory cytokines, supporting findings from in vitro studies. These results reinforce the cytoprotective and melanogenic effects of plant-derived phytocompounds and provide a preclinical basis for human trials.

#### 4.9.3. Clinical Trials and Case Reports

Clinical evidence, although limited by small sample sizes and heterogeneity, has demonstrated promising repigmentation outcomes with phytochemical interventions. Extracts of *Ginkgo biloba* administered orally for 6–12 weeks improved repigmentation in patients with non-segmental vitiligo, particularly when used as an adjunct to phototherapy [77,78].

Similarly, psoralen compounds from *Psoralea corylifolia* have been widely applied in combination with UVA or UVB phototherapy, enhancing melanogenesis and repigmentation (Curcumin from *Curcuma longa*, delivered topically or orally in small patient cohorts, has shown antioxidative and anti-inflammatory benefits with modest improvements in pigmentation [39].

Taken together, current clinical findings provide preliminary but meaningful support for the therapeutic potential of phytocompounds in vitiligo management, particularly when used as adjuncts to established treatments such as phototherapy. Importantly, the observed repigmentation outcomes are consistent with mechanistic evidence from in vitro and preclinical studies, including modulation of oxidative stress, suppression of inflammatory pathways, and stimulation of melanogenesis. However, the translational strength of these findings remains limited by methodological inconsistencies, including small cohort sizes, lack of standardized phytochemical formulations, and variability in treatment protocols.

In comparison with global evidence, plants such as *Ginkgo biloba*, *Curcuma longa*, and *Psoralea corylifolia* have been more extensively investigated. South African medicinal plants are underrepresented in experimental and clinical studies. This disparity highlights a significant research gap, particularly in the light of the region’s rich biodiversity and well-established ethnopharmacological knowledge systems [19].

## 5. Discussion

The mechanistic evidence synthesized in Section 4.4.1, Section 4.4.2, Section 4.4.3, Section 4.4.4, Section 4.4.5 and Section 4.4.6 demonstrates that plant-derived phytocompounds modulate a network of molecular pathways rather than acting through isolated targets, supporting the concept that effective vitiligo management requires integrated therapeutic strategies addressing multiple disease drivers simultaneously [36,78]. Within this network, oxidative stress functions as an upstream contributor that amplifies CD8^+^ T-cell–mediated cytotoxicity, with immune activation and melanogenic impairment representing converging downstream consequences that phytocompounds can simultaneously target [56]. This convergence across antioxidant, anti-inflammatory, and melanogenic pathways reflects systems-pharmacology models describing phytochemicals as regulators of interconnected signaling cascades rather than single-pathway agents and is particularly relevant in vitiligo, where oxidative stress, mitochondrial dysfunction, inflammatory signaling, and melanocyte apoptosis are mechanistically interdependent [23,78].

At the molecular level, NRF2-mediated antioxidant signaling functions as the central regulatory node linking redox homeostasis, mitochondrial integrity, inflammatory suppression, and melanocyte survival, while NF-κB and JAK/STAT represent the principal effector pathways through which phytocompounds simultaneously restore redox balance and attenuate IFN-γ–driven immune responses [43,55]. NRF2 activation, suppression of NF-κB and JAK/STAT, and concurrent regulation of MITF/tyrosinase signaling together produce coordinated antioxidant, anti-inflammatory, and melanogenic effects that exceed those of single-pathway interventions and explain the multi-target activity demonstrated by flavonoids, polyphenols, terpenoids, alkaloids, and psoralens [25,54,71].

Clinical and experimental observations from selected medicinal plants—including Ginkgo biloba, psoralen-containing preparations from Psoralea corylifolia, and curcumin derived from Curcuma longa—have demonstrated varying degrees of repigmentation efficacy and mechanistic activity [2,22,81]. These outcomes should be interpreted not as isolated compound-level effects but as part of a broader pattern of converging biological activity observed across diverse phytochemical classes [25]. Recent reviews of polyphenol-based interventions further support the concept that phytochemicals exert coordinated effects across oxidative stress, inflammatory signaling, melanogenic regulation, and mitochondrial protection in vitiligo [71]. Comparable multi-target behavior has also been documented in other inflammatory and oxidative-stress-associated disorders, supporting the broader translational relevance of phytochemical systems pharmacology [76]. Despite these promising findings, the clinical evidence base remains limited, and reported outcomes vary considerably due to differences in study design, extract composition, dosage, and treatment duration [10,22].

A primary barrier is poor bioavailability, which restricts the therapeutic efficacy of many plant-derived molecules, particularly polyphenols such as curcumin and resveratrol [81]. Variability in phytochemical composition across plant extracts further complicates standardization and reproducibility, and the limited number of well-designed randomized controlled trials, combined with short follow-up periods and small sample sizes, restricts the strength of current clinical conclusions [10,15]. Regulatory inconsistencies regarding herbal products also pose barriers to the development and approval of phytocompound-based interventions. Addressing these challenges requires a multidisciplinary and translational research approach integrating ethnopharmacology, molecular biology, pharmacology, and advanced drug-delivery systems. Future studies should prioritize the development of standardized, bioavailable formulations alongside mechanistically guided experimental designs incorporating validated melanocyte-based models and clinically relevant endpoints [15]. Recent translational advances involving nano-formulations, liposomal delivery systems, and targeted phytochemical encapsulation strategies have demonstrated improved compound stability, dermal penetration, and therapeutic efficacy in inflammatory skin disorders, suggesting potential applicability in vitiligo management [74,81]. Within this context, ethnobotanically prioritized species such as *Helichrysum odoratissimum* and Tagetes erecta represent valuable yet underexplored resources, and the under-representation of African medicinal plants in mechanistic dermatological research represents both a scientific limitation and a significant opportunity for phytopharmacological discovery [19,20,21].

Overall, the integration of mechanistic insight with clinical observations provides a strong foundation for advancing phytocompound-based interventions. The therapeutic relevance of phytocompounds in vitiligo lies in their capacity to simultaneously target multiple disease pathways—oxidative stress, inflammation, and impaired melanogenesis—supporting a shift toward integrative treatment strategies that complement existing therapies rather than replace them [23,25,77]. Continued progress will depend on bridging experimental findings with standardized and clinically translatable applications.

## 6. Knowledge Gaps and Research Limitations

Despite growing interest in plant-derived therapies for vitiligo, several important knowledge gaps and methodological limitations remain within the current body of literature. Addressing these issues is essential to strengthening the scientific evidence supporting phytocompounds as potential therapeutic agents for vitiligo.

One of the most notable limitations is the scarcity of detailed mechanistic studies investigating how phytochemicals influence melanocyte biology at the molecular level. While numerous studies report antioxidant or anti-inflammatory properties of plant extracts, relatively few provide comprehensive analyses of the specific signaling pathways involved in melanocyte survival, oxidative stress responses, or melanogenesis. Consequently, the precise molecular targets through which many phytocompounds exert their effects remain insufficiently characterized.

Another limitation relates to the restricted use of melanocyte-specific experimental models. Many studies evaluating plant-derived compounds rely on general cell lines or non-pigmentation biological systems, which may not accurately reflect the complex cellular environment of melanocytes in vitiligo. More extensive use of melanocyte cultures, vitiligo-relevant oxidative stress models, and advanced molecular assays would enhance the reliability and translational relevance of experimental findings.

A further gap in the literature is the underrepresentation of African medicinal plants, despite the region’s extensive biodiversity and rich ethnopharmacological traditions. While several plants from Asian and European traditional medical systems—such as *G. biloba*, *C. longa*, and *P. corylifolia*—have been relatively well studied, comparatively few investigations have explored the mechanistic potential of African plant species traditionally used for dermatological conditions. This imbalance highlights an important opportunity for future research aimed at validating and characterizing indigenous medicinal plants.

In addition, the lack of standardization in extract preparation and phytochemical characterization remains a significant challenge across many studies. Variability in plant sourcing, extraction methods, solvent systems, and phytochemical composition can lead to inconsistent experimental outcomes and limit reproducibility. Without standardized extraction protocols and clear identification of active constituents, it becomes difficult to compare findings across studies or translate experimental results into clinically applicable formulations.

Collectively, these limitations underscore the need for more rigorous experimental designs, improved model systems, and standardized phytochemical methodologies in future research. Addressing these gaps will be essential to advance the scientific validation of medicinal plants and facilitate the development of evidence-based phytotherapeutic approaches for vitiligo management.

## 7. Future Directions

Advancing the role of phytocompounds in vitiligo therapy will require a more integrative and mechanistically informed research framework that bridges ethnopharmacological knowledge with modern molecular and clinical approaches. Although existing studies highlight promising antioxidant, anti-inflammatory, and melanogenic activities of plant-derived compounds, future investigations should focus on addressing key methodological and translational challenges.

A primary priority is the development of hypothesis-driven mechanistic studies aimed at clarifying the molecular pathways through which phytochemicals influence melanocyte biology. Many current studies report phenotypic outcomes, such as increased melanin production or reduced oxidative stress, but lack detailed exploration of the signaling networks involved. Future research should incorporate advanced molecular approaches, including gene and protein expression analysis, pathway-specific assays, and systems biology techniques, to elucidate mechanisms related to oxidative stress modulation, immune regulation, and melanogenesis. Such studies will strengthen the biological plausibility of phytocompounds as therapeutic agents for vitiligo [25].

Another important direction involves the integration of ethnobotanical knowledge with molecular biology and pharmacological validation. Traditional medicinal systems provide valuable insights into plant species historically used for pigmentation disorders and dermatological conditions. Rigorous documentation and prioritization of these ethnobotanical leads by the broader research community can guide the selection of candidate plants for experimental investigation [73].

Combining this knowledge with modern analytical techniques, including metabolomic profiling, bioactivity-guided fractionation, and melanocyte-based assays, may facilitate the identification of novel bioactive compounds and therapeutic targets.

Improving standardization and reproducibility across studies is also critical for advancing phytocompound research. Variations in plant material sourcing, extraction procedures, and phytochemical composition can significantly influence experimental outcomes. Future work should emphasize standardized extraction protocols, accurate phytochemical characterization, and transparent reporting of experimental conditions. These practices will enhance comparability between studies and support the development of consistent, quality-controlled plant-derived formulations.

Finally, greater attention must be given to the translational and clinical relevance of experimental findings. While numerous in vitro studies demonstrate promising biological activity, relatively few compounds progress to well-designed clinical investigations. Bridging this gap will require interdisciplinary collaboration between ethnopharmacologists, molecular biologists, pharmacologists, and clinicians. Carefully designed preclinical studies, followed by standardized clinical trials evaluating safety, efficacy, and long-term outcomes, will be essential to establish the therapeutic potential of phytocompounds for vitiligo management.

Overall, integrating mechanistic research, ethnobotanical insights, standardized methodologies, and translational clinical studies may pave the way for the development of evidence-based phytotherapeutic strategies capable of complementing existing treatments and improving outcomes for individuals affected by vitiligo.

## 8. Conclusions

This review synthesizes current mechanistic and translational evidence supporting the therapeutic potential of medicinal plant-derived compounds for the treatment of vitiligo. Phytochemicals, including flavonoids, polyphenols, terpenoids, and psoralens from species such as *Ginkgo biloba*, *Curcuma longa*, and *Psoralea corylifolia*, demonstrate antioxidant, anti-inflammatory, immunomodulatory, cytoprotective, and melanogenesis-promoting properties that target interconnected pathways governing redox homeostasis, immune activation, cellular survival, and pigment production. When combined with phototherapeutic approaches, these compounds have shown encouraging biological activity and, in some cases, clinical benefit. NRF2-mediated redox regulation emerges as a critical therapeutic node intersecting with NF-κB and JAK/STAT signaling, supporting melanocyte survival and melanogenic activity, thereby positioning multi-target phytocompounds as rational candidates for vitiligo therapy.

Despite these promising findings, translational progress remains constrained by variability in plant materials and extraction methods, inadequate phytochemical standardization, insufficient mechanistic characterization, and a scarcity of well-designed clinical studies. Furthermore, the under-representation of African medicinal plants in mechanistic and translational research highlights an important knowledge gap, particularly given their extensive ethnobotanical use and rich phytochemical diversity.

Future research should therefore prioritize three coordinated actions: standardized phytochemical characterization, pathway-focused mechanistic investigations, and robust preclinical and clinical studies that evaluate integrated redox-, immune-, and pigmentation-related outcomes. In parallel, advances in formulation science, including nanoformulations, liposomal delivery systems, and targeted phytochemical encapsulation technologies, may improve bioavailability, stability, and therapeutic efficacy, thereby facilitating clinical translation.

Overall, the available evidence reviewed supports a systems-level model in which phytocompounds act on interconnected networks linking oxidative stress, inflammation, melanocyte survival, and melanogenesis. Continued integration of ethnopharmacological knowledge with modern molecular and translational approaches may accelerate the identification of novel phytotherapeutic agents and contribute to the development of more effective, mechanism-based interventions for vitiligo management.

## Figures and Tables

**Table 1:** Medicinal plants and bioactive compounds with potential therapeutic relevance in vitiligo: experimental models, biological activities, mechanisms of action, and principal outcomes.

Medicinal Plant	Major Bioactive Compounds	Study Type/Experimental Model	Biological Activity/Proposed Mechanisms of Action	Principal Findings/Key Outcomes	References
*Ginkgo biloba* L. *(Maidenhair tree)*	Flavonoids (quercetin, kaempferol), terpene lactones (ginkgolides, bilobalide)	Clinical studies in vitiligo patients; in vitro oxidative stress models	Antioxidant activity through enhancement of cellular redox defense (including NRF2-related pathways); inhibition of NF-κB-mediated inflammatory signaling; protection against oxidative melanocyte injury	Clinical evidence suggests reduced disease progression and partial repigmentation in some patients; mechanistic studies indicate improved melanocyte survival under oxidative stress	[45,46]
*Curcuma longa* L. *(Turmeric)*	Curcumin (polyphenolic diarylheptanoid)	In vitro melanocyte models; experimental oxidative stress models; small clinical investigations	ROS scavenging; modulation of inflammatory mediators; suppression of NF-κB signaling; regulation of melanogenic pathways, including MITF-associated signaling	Demonstrated reduction in oxidative damage, improved cellular antioxidant capacity, and potential enhancement of melanocyte viability	[39,44]
*Psoralea corylifolia* L. *(Bakuchi)*	Psoralens (psoralen, isopsoralen), flavonoids	Clinical application with PUVA/UV phototherapy; melanogenesis-related experimental studies	Photosensitization leading to UV-induced melanogenesis; stimulation of tyrosinase activity and melanin synthesis	Combination with phototherapy promotes repigmentation; supports melanocyte activation and restoration of pigmentation	[47,48]
*Helichrysum odoratissimum* (L.) *Sweet (Impepho)*	Flavonoids, terpenoids, phenolic compounds	In vitro biochemical studies; emerging mechanistic investigations	Antioxidant activity; reduction in oxidative stress burden; potential melanocyte cytoprotection through improved redox homeostasis	Preliminary evidence indicates possible protection of melanocytes against oxidative injury; further vitiligo-specific studies required	[22,49]
*Tagetes erecta* L. *(African marigold)*	Carotenoids, flavonoids, terpenoids	Preliminary in vitro cellular investigations	Antioxidant and anti-inflammatory activity; possible modulation of melanogenesis-related pathways	Bioactive constituents show potential melanogenic and cytoprotective effects, suggesting possible relevance for melanocyte preservation	[50,51]
*Camellia sinensis* (L.) *Kuntze (Green tea)*	Catechins (EGCG, ECG, EGC)	In vitro cellular models; oxidative stress studies	Antioxidant activity; modulation of inflammatory responses; regulation of immune-mediated oxidative damage	Catechins reduce oxidative stress markers and may enhance melanocyte resistance to environmental stressors	[21,52]
*Vitis vinifera* L. *(Grape)*	Resveratrol, proanthocyanidins, flavonoids	Cellular oxidative stress models	Antioxidant activity; anti-inflammatory effects; regulation of mitochondrial function and cellular stress responses	Improves cellular resilience by reducing oxidative damage and supporting protective stress-response pathways	[22,43]

Notably, flavonoid- and polyphenol-rich plants dominate the evidence base, reinforcing the central role of antioxidant and anti-inflammatory mechanisms in melanocyte protection. Abbreviations: NRF2, nuclear factor erythroid 2-related factor 2; NF-κB, nuclear factor kappa B; ROS, reactive oxygen species; PUVA, psoralen plus ultraviolet A.

## Data Availability

No new data was generated or analyzed in support of this research. All information presented in this review article is derived from previously published studies, which are appropriately cited within the manuscript.

## Abbreviations

The following abbreviations are used in this manuscript:

AI	Artificial Intelligence
Akt	Protein Kinase B
ARE	Anti-oxidant Response Element
bFGF	Basic Fibroblast Growth Factor
CD8	Cluster Differentiation 8
CXL	Chemokine Ligand
DAMP	Damage-Associated Molecular Patterns
ET-1	Endothelin-1
EGCG	Epigallocatechin gallate
GPX/GPX4	Glutathione Peroxidase
H_2_O_2_	Hydrogen Peroxide
IFN-γ	Interferon
IL-6	Interleukin 6
JAK	Janus Kinase
MAPK	Mitogen-Activated Protein Kinase
miRNA	Micro RNA
MITF	Microphthalmia-Associated Transcription Factor
MSH	Melanocyte Stimulating Hormone
NB-UVB	Narrowband Ultraviolet B
NF-κB	Nuclear Factor-Kappa B
NRF2	Nuclear Factor Erythroid 2-Related Factor 2
PI3K	Phosphatidylinositol 3-Kinase
PICO	Population–Intervention–Comparator–Outcome
PRISMA	Preferred Reporting Items for Systematic Reviews and Meta-Analyses
PUVA	Psoralen–Ultraviolet A
qPCR	Quantitative PCR
RCTs	Randomized Controlled Trials
ROS	Reactive Oxygen Species
RTK	Receptor tyrosine kinase
SCF	Stem Cell Factor
STAT	Signal Transducer and Activator of Transcription
T. Cells	Thymus Lymphocytes
TNF-α	Tumor Necrosis Factor Alpha
T RM	Resistant Memory T
TRP-1	Tyrosinase-Related Protein-1
TRP-2	Tyrosinase-Related Protein-2
TYR	Tyrosinase
α-Pinene	Alpha Pinene
β-Pinene	Beta Pinene
